# Tailored Exercise Training Counteracts Muscle Disuse and Attenuates Reductions in Physical Function in Individuals With Amyotrophic Lateral Sclerosis

**DOI:** 10.3389/fphys.2019.01537

**Published:** 2019-12-26

**Authors:** Alessandra Ferri, Francesca Lanfranconi, Giovanni Corna, Riccardo Bonazzi, Samuele Marchese, Andrea Magnoni, Lucio Tremolizzo

**Affiliations:** ^1^Institute for Health and Sport, Victoria University, Melbourne, VIC, Australia; ^2^Foundation Monza and Brianza for the Mother and Her Child, Monza, Italy; ^3^School of Medicine and Surgery, University of Milano-Bicocca, Monza, Italy; ^4^Italian Academy of Osteopathic Medicine (AIMO), Saronno, Italy; ^5^Resting Home “San Pietro,” Cooperativa La Meridiana, Monza, Italy; ^6^Neurology Unit, San Gerardo Hospital, Monza, Italy

**Keywords:** amyotrophic lateral sclerosis, exercise training, aerobic capacity, muscle strength, physical function

## Abstract

Amyotrophic lateral sclerosis (ALS) is a neurodegenerative disease, characterized by the progressive loss of motor neurons, which leads to a reduction in strength and exercise capacity. Although the concept of “Exercise is Medicine” is accepted for many diseases, the role of exercise in individuals with ALS is still debated. The aim of this study was to propose a tailored exercise training program that was both safe and effective for individuals with ALS, and to evaluate the effects of this combined, moderate-intensity, aerobic and strength training program on aerobic capacities, strength, and physical function. Sixteen individuals with ALS were randomly assigned to either a training (three times a week for 12 weeks; TRAIN, *n* = 8) or usual care (continued their usual standard of care and served as control; UC, *n* = 8) group. Peak power, peak oxygen uptake, as well as the gas exchange threshold (GET) during a cardiopulmonary exercise test (CPET) on a cycle ergometer, and the maximal strength (1RM) of the knee extensor muscles, were evaluated before and after 12 weeks. Participants also performed the “Timed Up and Go” (TUG) and the “6-min walking” (6MWT) tests. The ALS Functional Rating Scale revisited (ALSFRS-R), the ALS Severity Scale (ALS-SS), and the McGill quality of life (QoL) questionnaire were also measured. The GET increased from 0.94 ± 0.08 to 1.06 ± 0.10 L min^–1^ in TRAIN (*p* = 0.009) and decreased from 0.79 ± 0.17 to 0.72 ± 0.17 L min^–1^ in UC (*p* = 0.001). There was a significant difference between groups for changes in TUG (9.1 ± 5.5% improvement in TRAIN and 56.8 ± 18.5% worsening in UC, *p* = 0.002), ALSFRS-R (4.7 ± 2.6% decrease in TRAIN and 23.0 ± 5.6% decrease in UC, *p* = 0.007), and for the ALS-SS (2.2 ± 2.1% decrease in TRAIN and 12.4 ± 4.4% decrease in UC, *p* = 0.04). Even if the 1RM of the knee-extensor muscles showed a tendency to increase in TRAIN (70.1 ± 30.0%, *p* = 0.07), there was not a statistically significant difference (*p* = 0.57) with respect to the changes in the UC group (44.9 ± 20.7% increase, *p* = 0.11). This study showed that a combined moderate-intensity aerobic and strength training program, tailored to the physical capacities of each individual, can improve aerobic fitness and maintain physical function in individuals with ALS.

## Introduction

Amyotrophic lateral sclerosis (ALS) is a neurodegenerative disease characterized by the progressive degeneration and death of upper and lower motor neurons, which leads to a reduction in muscle mass and strength and ultimately death. Patients with ALS have a reduced exercise tolerance (Mezzani et al., 2012; Lanfranconi et al., 2017), which is associated with impaired physical function and quality of life (QoL; Korner et al., 2015). Currently there is no cure for this disease, and the lack of effective treatments able to alter the pathophysiological pathways that modulate disease progression has led to suggestions that the muscle may be a strategic therapeutic target (Shefner, 2009), in order to counteract the inevitable loss of function in patients with ALS. Increasing the strength and the oxidative capacity of muscle fibers whose innervation is intact could preserve function in these patients and potentially also impact survival rates. Due to the well-known beneficial effects of exercise in improving muscle strength and oxidative capacity in healthy individuals, tailored exercise training could represent a possible therapeutic intervention in patients with ALS.

Even if exercise has been scientifically proven to be beneficial in maintaining/increasing aerobic fitness, physical function, and independence in a wide range of clinical conditions, such as cancer (Iyengar and Jones, 2019), as well as neurodegenerative (Motl and Pilutti, 2012; LaHue et al., 2016), muscle (Voet et al., 2013), and cardiovascular (Pryzbek et al., 2019) diseases, the role of physical activity in patients with ALS is still debated. As a result, patients with ALS are usually advised to avoid or reduce their physical activity, which can further exacerbate the decrease in exercise tolerance and muscle strength due to cardiovascular deconditioning and muscle disuse, leading to a deleterious cycle of reduced function. Recently, a few published studies have shown the tolerability and safety of an exercise regimen in patients with ALS (Clawson et al., 2018; Merico et al., 2018), and the positive effects exercise has in reducing the global function decline evaluated by disease-specific rating scales (Drory et al., 2001; Dal Bello-Haas et al., 2007; Lunetta et al., 2016). While these results are encouraging, this evidence is not sufficiently detailed to recommend a specific exercise training program. In particular, there is a lack of data on the effects of exercise on objective physiological outcomes, such as maximal aerobic capacity and muscle strength.

The increasing recognition of “Exercise is Medicine” highlights the need to determine the exact type and dose of exercise to improve a specific physical capacity for a specific population. The main challenge when prescribing an exercise training program for patients with ALS is to find the right balance between an exercise that is both safe and effective. In this study, our purpose was to propose exercises that provided a sufficient stimulus to increase muscle aerobic capacity and strength, with minimal or no muscle damage, and without fatiguing the muscle and the patients. Strength training, in particular if proposed between a moderate and a high intensity (around 60–80% of 1 Repetition Maximum, 1RM) and in sets of 6–12 repetitions (Kraemer et al., 1996), has the main benefit to increase muscle mass and strength. Aerobic training at a moderate intensity (near the anaerobic threshold, the point where the lactate starts to be produced and fatigue appears) represents the optimal intensity for improvement of endurance fitness (resulting from an increase in the oxygen uptake at the anaerobic threshold; Jones and Carter, 2000) and can have a beneficial effect on exercise tolerance and fatigue during daily living activities. In the light of these observations, our strength training was set at the lower effective intensity (60% of 1RM) and the aerobic training below the anaerobic threshold (80% of the anaerobic threshold), so that the patients could exercise without producing lactate and with less fatigue.

The purpose of this study was to gain knowledge on the effects of a 12-week combined aerobic and strength training program at a moderate intensity in individuals with ALS on muscle strength, aerobic capacity, and physical function. We hypothesized that the individuals with ALS who exercised would present a reduced decline in muscle strength and aerobic capacity by counteracting muscle disuse. We hypothesized also that the reduced impairment of these characteristics would translate to a reduced deterioration of their physical function and QoL.

## Materials and Methods

### Participants

Sixteen individuals with a diagnosis of possible, probable, or definite ALS (according to the revised El Escorial criteria; Brooks et al., 2000) were recruited through four Italian neurological centers specialized in the treatment of individuals with this disease. A neurological assessment was made in order to verify the patient’s eligibility for this project and to stratify the phenotype: patients with a time since diagnosis of less than 48 months and able to pedal on a cycle ergometer were included in the study. All participants underwent a medical evaluation to exclude the presence of any acute cardiopulmonary and/or infectious diseases. A careful collection of their physical activity history was also recorded. Most participants (70%) were on riluzole therapy (100 mg daily) for an average time of 6 months, and continued the medication for the entire intervention period. Participants were assigned randomly based on age, sex, BMI and time elapsed from diagnosis to two groups: (1) training (TRAIN, *n* = 8, 2 bulbar onset; 2 females), who conducted a tailored exercise training program three times per week for 12 weeks; (2) usual care (UC, *n* = 8, 3 bulbar onset; 2 females), who received passive manual therapy once weekly/fortnight for 12 weeks, and who served as a control group. Table 1 shows the demographic and clinical characteristics of patients in both the TRAIN and UC groups. Patients in both groups were not significantly different for age, time from diagnosis, BMI, fat free mass or peak oxygen uptake (V̇O_2__peak_). The V̇O_2__peak_, which can be considered an objective measure of exercise tolerance in patients with ALS (Lanfranconi et al., 2017) and the motor ALS functional rating scale revised version (ALSFRS-R) sub-score were not statistically different between the two groups. The ALSFRS-R is a validated rating instrument to monitor the progression of disability in patients with ALS, evaluating three different domains (bulbar, motor, and respiratory function) (Cedarbaum et al., 1999). There was a tendency toward a significant difference for the total ALSFRS-R scores, probably because in the UC group there was a greater proportion of the bulbar sub-type (3 vs. 2) that impacted on the total score. Patients with a bulbar onset develop initial symptoms in the bulbar-innervated muscles that control speech and swallowing, and present the worst survival rate (Chio’ et al., 2011). The higher proportion of patients with bulbar onset could have also sped up the disease progression of the UC group, with a quicker physical deterioration, with respect to the TRAIN group.

**TABLE 1:** Demographic and clinical characteristics of patients with amyotrophic lateral sclerosis (ALS) in the training (TRAIN) and usual care (UC) groups before the 12-week intervention (T0).

	**TRAIN (*n* = 8)**	**UC (*n* = 8)**	***p***
Age (years)	50.7 ± 3.3	55.5 ± 5.95	0.33
Sex (♀/♂)	2/6	2/6	
Onset (B/S)	2/8	3/8	0.30
^∗^Duration (months)	20.5 ± 20.3	13.4 ± 6.6	0.36
BMI (kg m^–2^)	25.2 ± 3.4	26.0 ± 2.8	0.63
FFM (kg)	60.9 ± 5.12	59.1 ± 4.9	0.75
NIV (no/yes)	6/2	5/3	0.59
ALSFRS-R	40.4 ± 1.5	35.0 ± 3.4	0.09
ALSFRS-R motor	16.9 ± 1.3	13.8 ± 2.5	0.16
V̇O_2__peak_ (mL kg^–1^ min^–1^)	21.3 ± 2.2	17.3 ± 2.1	0.16

*Values are mean ± SE. BMI = body mass index; FFM = fatty free mass; onset B = bulbar or S = spinal; NIV = non-invasive ventilation; ALSFRS-R = ALS functional rating scale revised; V̇O_2__*peak*_ = peak oxygen uptake. ^∗^From diagnosis to start of study.*

Participants were informed about the risks of testing and training and provided informed consent to participate in the study. The study conformed to the standards set by the latest revision of the Declaration of Helsinki. The procedures were approved by the ethics committee of the University of Milano-Bicocca (protocol #129, 10-June-2014, University of Milano-Bicocca). Personal data were treated according to standard principles of confidentiality. The data presented in this manuscript are part of the “ME&SLA” project (ClinicalTrials.gov Identifier: NCT02548663).

### Experimental Design

At baseline (T0) and after 12 weeks of training or standard of care (T1) each participant underwent the following assessments:

• Cardiopulmonary exercise test (CPET) on a cycle ergometer (Monark-LC6: Monark, Varberg, Sweden) under medical supervision and with 12-lead electrocardiography monitoring (Quark C12x: COSMED, Roma, Italy). After 2 min of unloaded cycling, the power was increased every minute by step increments of 3, 5, or 15 W, depending on the fitness level of each subject, and participants were encouraged to exercise until exhaustion. Exhaustion was defined as the inability to maintain the pedaling frequency (60 revolutions min^–1^, RPM), despite vigorous encouragement by the experimenters. Pulmonary ventilation (V̇E, in BTPS), O_2_ consumption (V̇O_2_, in STPD) and CO_2_ output (V̇CO_2_, in STPD) were determined breath-by-breath by a computerized metabolic cart (Vmax SPECTRA 229: SensorMedics Corporation Yorba Linda, Yorba Linda, CA, United States). Expiratory flow was recorded at the mouth of the subject by a mass flow sensor (hot wire anemometer). V̇O_2_ and V̇CO_2_ were determined through continuous monitoring of PO_2_ and PCO_2_ at the mouth throughout the respiratory cycle and from established mass balance equations. The gas exchange threshold (GET), which represents the transition from aerobic metabolism to anaerobic (with consequent lactate production) plus aerobic metabolism, was calculated by conventional methods (Beaver et al., 1986). Arterial blood O_2_ saturation (SaO_2_) was checked continuously through pulse oximetry at the finger (RAD 9 Signal Extraction Pulse Oximeter: Masimo Corporation, Irvine, CA, United States). The environmental temperature during exercise was standardized to 20°C using an air-conditioning system and the current barometric pressure was recorded. The rating of perceived exertion (RPE) was obtained through the Borg scale. Blood lactate was determined on capillary blood samples obtained from an ear lobe, at rest and at 1, 3, 5, 7 min after the termination of CPET by a dual-channel analyzer (BIONSEN C-line, EKF Diagnostics, Cardiff, United Kingdom). The highest value obtained during the 7 min of recovery was considered the peak lactate concentration (La^+^_peak_).
• The “Timed Up and Go” Test (TUG; Podsiadlo and Richardson, 1991) and the 6-min Walking Test (6MWT, Guyatt et al., 1985).
• Maximal strength of the quadriceps muscles by a ten repetition maximum (10RM) test during a bilateral leg extension (LE) exercise performed on an isotonic machine (Leg Extension Alpha pro, multi-function bench, Kettler, Ense, Germany). The value obtained was then used to estimate the 1RM (Brzycki, 1993) – an indication of the maximal strength of the quadriceps muscle.
• ALS functional rating scale-revised version (ALSFRS-R, Cedarbaum et al., 1999). The ALSFRS-R includes 12 questions, which are rated on a five-point scale from 0 = can’t do to 4 = normal ability. Individual scores are summed to produce a total score between 0 = worst and 48 = best.
• ALS-SS (Hillel et al., 1989) is a complimentary scale of ALSFRS-R, and has the same purpose to assess function in four categories (speech, swallowing, lower- and upper-extremity abilities).
• McGill Quality of Life Questionnaire (Cohen et al., 1995), to assess the QoL of patients with a life-threatening illness.
• Body fat-free mass (FFM) by measuring skinfolds at seven sites (C10Plicometer Tanner – Whitehouse; Holtain, Ltd., Crymych, United Kingdom), and applying the Jackson Pollock body density equation (Jackson and Pollock, 1978).

### Training Program

Participants in the TRAIN group visited a gym located at Clinica S. Pietro, La Meridiana, Monza, Italy, three times/week for 12 weeks. Each session, of 60 min duration, was supervised by two sport scientists, a medical doctor, and a student from Medical School; there was a patient/therapist ratio of 1:1. The training program was characterized by aerobic, resistance, balance, and stretching exercises, distributed as follows:

• 15 min of cycling at an intensity corresponding to 80% between baseline and the GET calculated during CPET.
• 25 min of strength exercises at an intensity corresponding to 60% of 1RM. Three sets of 10 repetitions (2 min rest between sets) of upper (biceps curl and arm lateral raise) and lower (squat, calf raise, and LE) body exercises were performed with dumbbells; the LE exercise was performed on an isotonic machine. TheraBand^TM^ elastics were also used to perform chest press and seated row exercises. Strength exercises were alternatively performed during the week. To reduce the possibility of muscle damage, the eccentric phase of the exercises was avoided.
• 10 min of proprioceptive exercises, most of which were performed on the BOSU^®^ Pro balance trainer.
• 10 min of upper and lower extremity stretching exercises realized on a Pancafit^®^.

Safety parameters such as blood pressure, heart rate, and oxygen saturation, as well as training intensity and attendance at training sessions, were documented in a patient diary.

After 6 weeks of training the 10RM test was repeated to adjust the load to the new values obtained, so the load of 60% of 1RM was maintained throughout the training. A 200-ml hyperproteic supplement was given immediately after each training session (300 kcal, 12 g of proteins, 37 g of carbohydrates, 12 g of fat, 0.1 g of fibers, and 12 g of minerals) along with 200 ml of water (eventually thickened).

Patients randomized to the UC group were instructed to maintain their usual daily activities. They were given the same supplement as the participants in the TRAIN group three times/week for 12 weeks.

### Statistical Analysis

Values are expressed as mean ± Standard Error (SE). Effect size (ES) was estimated with Cohen’s *d*. A Shapiro–Wilk test was performed to test for normality of the data. The statistical significance of the difference in mean values between groups (TRAIN vs. UC) was evaluated by paired two-tailed Student’s *t*-test, while the difference in the proportion of drop-outs, non-invasive ventilation (NIV) and disease onset between groups was calculated by a two proportion *z*-test. Regression and correlation analyses were performed using the least squared residuals method. The level of significance was set at *p* < 0.05, and tendency was noted for 0.05 < *p* < 0.09. All statistical analyses were performed using a commercially available software package (Prism 6.0: GraphPad, La Jolla, CA, United States).

## Results

### Participation

**FIGURE 1:**
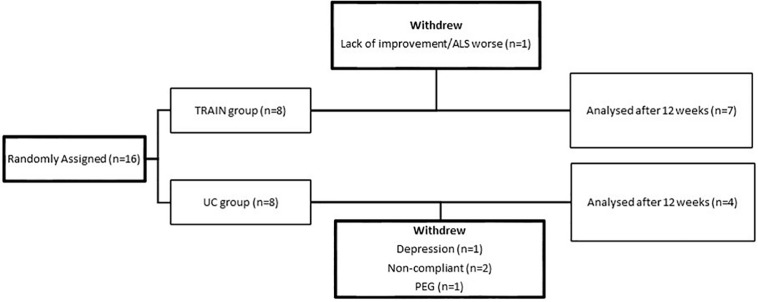
Flow chart of participants from random assignment to interventions, withdrawals, and completion of the project.

The flow chart (Figure 1) shows the drop-out rate for both groups (*p* = 0.10), with 1 drop-out for the TRAIN group (one patient showed the first signs of depression and felt that exercise was not good for him), and four drop-outs for the UC group (one patient required percutaneous endoscopic gastrostomy at the time of the final test evaluation; two found it difficult to come to the hospital for the test sessions, and one had severe depression). In the TRAIN group the adherence to the training program was 84.8 ± 6.3%. The satisfaction regarding the training program, evaluated by the Visual Analog Scale (VAS), was 8.8 ± 0.8. No adverse events happened during the 12 weeks of training.

### Cardiopulmonary Exercise Testing (CPET)

All participants terminated the CPET due to their inability to maintain the pedaling frequency of 60 RPM. The respiratory exchange ratio (RER) and [La^+^] values at the end of CPET were 1.12 ± 0.02 and 4.3 ± 0.6 mmol L^–1^ for TRAIN, and 1.13 ± 0.07 and 4.4 ± 1.1 mmol L^–1^ for UC. The TRAIN and UC groups reached 81.2 ± 2.9 and 80.2 ± 6.4% of the age-predicted maximum HR, respectively.

**FIGURE 2:**
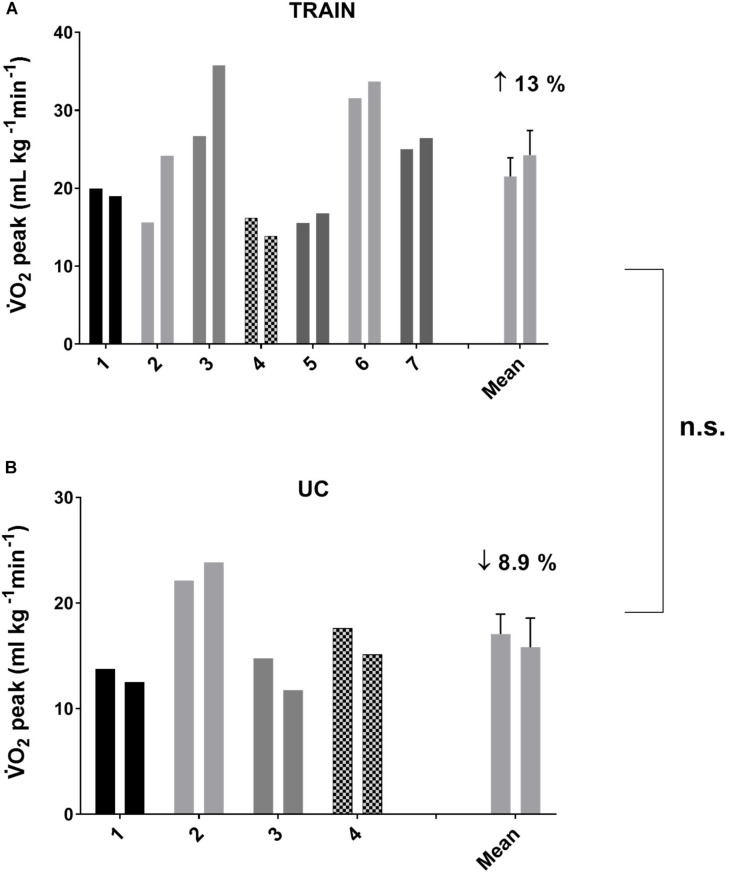
Individual and average values for peak oxygen uptake (V̇O_2peak_) before and after 12 weeks of exercise training (**A**, TRAIN) and usual care (**B**, UC). Average data are expressed as mean + SE. n.s., not significantly different between TRAIN and UC.

In TRAIN, the GET increased significantly from 0.94 ± 0.08 at T0 to 1.06 ± 0.10 L min^–1^ at T1 (*p* = 0.009, ES = 0.47), while in UC the GET decreased significantly from 0.79 ± 0.17 at T0 to 0.72 ± 0.17 L min^–1^ at T1 (*p* = 0.001, ES = 0.19), indicating that the aerobic training produced an improved exercise tolerance in individuals with ALS. In TRAIN, V̇E_peak_ showed a tendency to increase from 62.32 ± 10.44 to 78.64 ± 14.91 L min^–1^ (27.3 ± 11.5%, *p* = 0.09, ES = 0.49), while there was no change in V̇E_peak_ for the UC (−6.3 ± 4.0%, *p* = 0.29, from 49.17 ± 8.61 to 45.53 ± 7.78 L min^–1^). We also found significant between groups differences in percentage for changes in GET, and a tendency toward a change for *W*_peak_ (*p* = 0.08) and V̇E_peak_ (*p* = 0.09). Despite a 13.0% increase in V̇O_2__peak_ in TRAIN (with 5 out of 7 patients improving their value) and an 8.9% decrease in UC (with 3 out of 4 participants worsening their value), we did not find significant group differences for the change in V̇O_2__peak_ (Figure 2). Even if the differences were not statistically significant for most of the parameters evaluated (with the exception of the GET), due in part to the underpowered samples and the differential dropout rates, it is interesting to note the different direction of the arrows (indicating the changes) in the two groups (Table 2).

**TABLE 2:** Mean values before (T0) and after (T1) 12 weeks of exercise training (TRAIN) and usual care (UC), and percentage changes of different variables measured during a cardiopulmonary exercise test (CPET).

	**TRAIN**	**TRAIN**	**TRAIN**	**UC**	**UC**	**UC**
	**T0**	**T1**	**% Change**	**T0**	**T1**	**% Change**
*W*_peak_ (W)	111.1 ± 19.0	117.6 ± 23.2	↑ 3.0 ± 5.4	70.2 ± 13.3	59.7 ± 19.7	↓ 20.12 ± 10.4 $
			ES = 0.12			ES = 0.28
FFM (kg)	59.6 ± 5.1	59.9 ± 4.4	↑ 1.3 ± 2.0	59.8 ± 4.9	58.5 ± 4.7	↓ 2.0 ± 1.1
			ES = 0.02			ES = 0.12
*W*_peak_/FFM (W kg^–1^)	1.77 ± 0.2	1.85 ± 0.3	↑ 2.1 ± 5.2	1.15 ± 0.14	1.00 ± 0.24	↓ 17.9 ± 9.4
			ES = 0.12			ES = 0.30
V̇O_2__peak_ (mL kg^–1^ mm^–1^)	21.5 ± 2.4	24.3 ± 3.1	↑ 13.0 ± 8.4	17.1 ± 1.9	15.8 ± 2.8	↓ 8.9 ± 5.4
			ES = 0.37			ES = 0.27
**GET (L min**^–^**^1^)**	**0.94 ± 0.08**	**1.06 ± 0.10^∗^**	↑ **11.9 ± 3.6**	**0.79 ± 0.17**	**0.72 ± 0.17^∗^**	↓ **19.9 ± 3.4#**
			**ES = 0.47**			**ES = 0.19**
V̇E_peak_ (L min^–1^)	62.3 ± 10.4	78.6 ± 14.9	↑ 27.3 ± 11.5	49.2 ± 8.6	45.5 ± 7.8	↓ 6.3 ± 4.0 $
			ES = 0.49			ES = 0.20
La^+^_peak_ (mmol L^–1^)	4.3 ± 0.6	4.6 ± 1.0	↑ 4.3 ± 14.4	4.4 ± 1.1	3.9 ± 1.0	↓ 11.6 ± 4.6
			ES = 0.15			ES = 0.22

*Values are expressed as mean ± SE. *W*_*peak*_ = peak power output; FFM = fatty free mass; V̇O_2__*peak*_ = peak oxygen uptake; GET = gas exchange threshold; V̇E_*peak*_ = peak ventilation; La^+^_*peak*_ = peak lactate concentration. ^∗^*p* < 0.05 statistically different from T0. ^#^*p* < 0.05 statistically different from TRAIN. ^$^0.05 < *p* < 0.09 tendency to a difference from TRAIN. Only the row indicated with bold is statistically significant between T0 and T1 for both groups.*

### Physical Function

**FIGURE 3:**
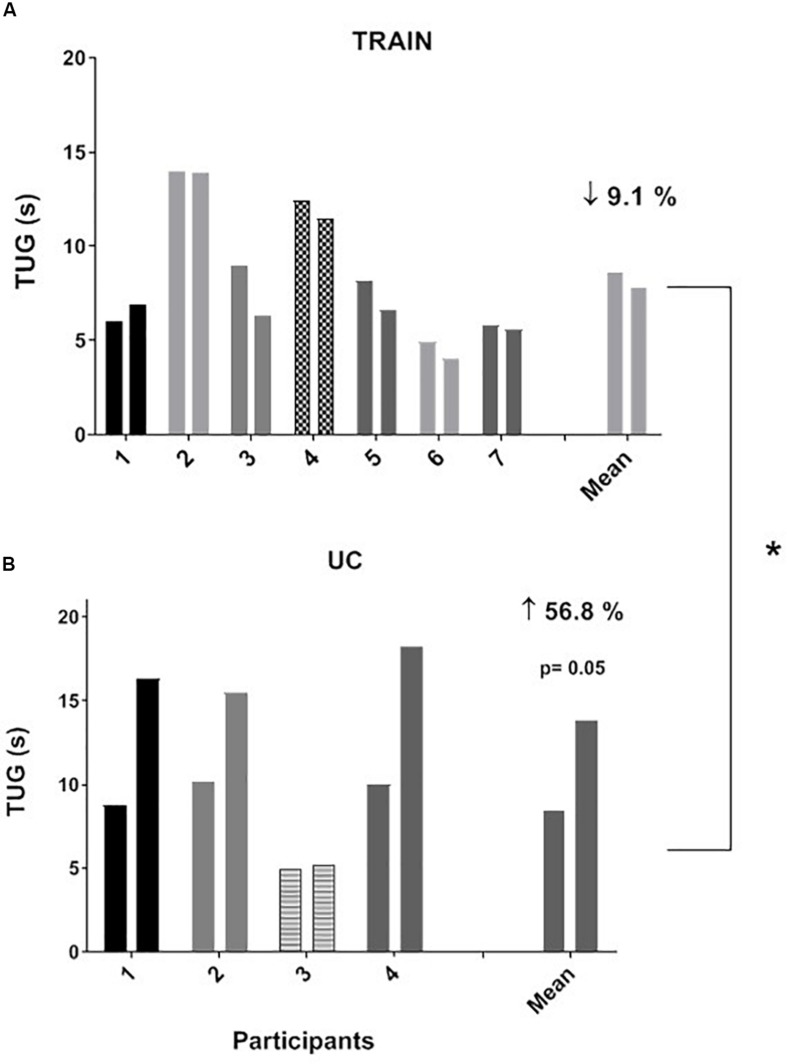
Individual and average values for “Timed Up and Go” test (TUG) before and after 12 weeks of exercise training (**A**, TRAIN) and usual care (**B**, UC). Average data are expressed as mean + SE. ^∗^Significantly different between TRAIN and UC, *p* < 0.05.

**FIGURE 4:**
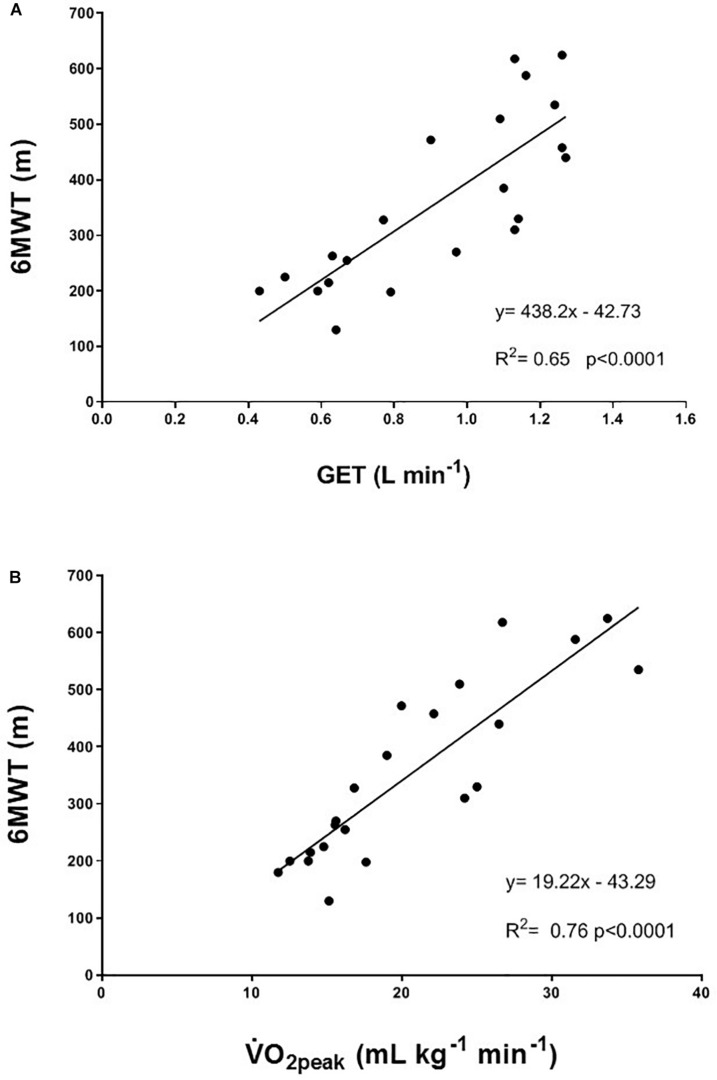
Correlation between **(A)** gas exchange threshold (GET) and the 6-min walking test (6MWT); and **(B)** peak oxygen uptake (V̇O_2peak_) and the 6-min walking test (6MWT). Participants from both the TRAIN and UC groups are considered in the correlation.

The individual values for the TUG test are shown in Figure 3. In the TRAIN group, even if 6 of 7 patients performed the TUG test at T1 faster than at T0, the mean group change was not significantly different between T0 and T1 (mean reduction of time of 9.1 ± 5.5%, *p* = 0.12, ES = 0.22). In the UC group, 4 out of 4 patients showed an impaired performance in the TUG test (increase in time of 56.8 ± 18.5%, *p* = 0.05, ES = 1.29). The percentage changes between TRAIN and UC were significantly different (*p* = 0.002). The TRAIN and UC groups did not differ significantly at T0 and T1 in the distance covered during the 6MWT, with a small non-significant increase at T1 in TRAIN (+4.5 ± 7.9%) and decrease in UC (−10.7 ± 10.2%). When the data of both groups were plotted together, there was a significant correlation between the GET and the 6MWT (Figure 4A), indicating that the individuals that had higher GET values had a better performance in the 6MWT. A similar correlation was also found between V̇O_2__peak_ and 6MWT (Figure 4B).

### Body Fat-Free Mass (FFM) and Strength Measurements

There was no statistical difference (*p* = 0.18) between changes in FFM in TRAIN (+1.4 ± 2.0%) and in UC (−2.0 ± 1.1%). While in TRAIN the power expressed relative to the metabolically active body mass (W_peak_/FFM in W kg^–1^) was maintained after the 12 weeks of training (+2.1 ± 5.2%, *p* = 0.4), in UC this value was reduced (even if not significantly, *p* = 0.46) by 17.9 ± 9.4%. In TRAIN, 1RM LE showed a tendency to increase from 39.8 ± 8.2 to 63.8 ± 16.2 kg (70.1 ± 30.0%, *p* = 0.07, ES = 0.74) without showing a significant difference (*p* = 0.57) with respect to the changes in the UC group (+44.9 ± 20.7% from 36.8 ± 8.9 to 53.2 ± 13.4 kg, *p* = 0.11).

### ALSFRS-R and ALS-SS Scores and Quality of Life

**FIGURE 5:**
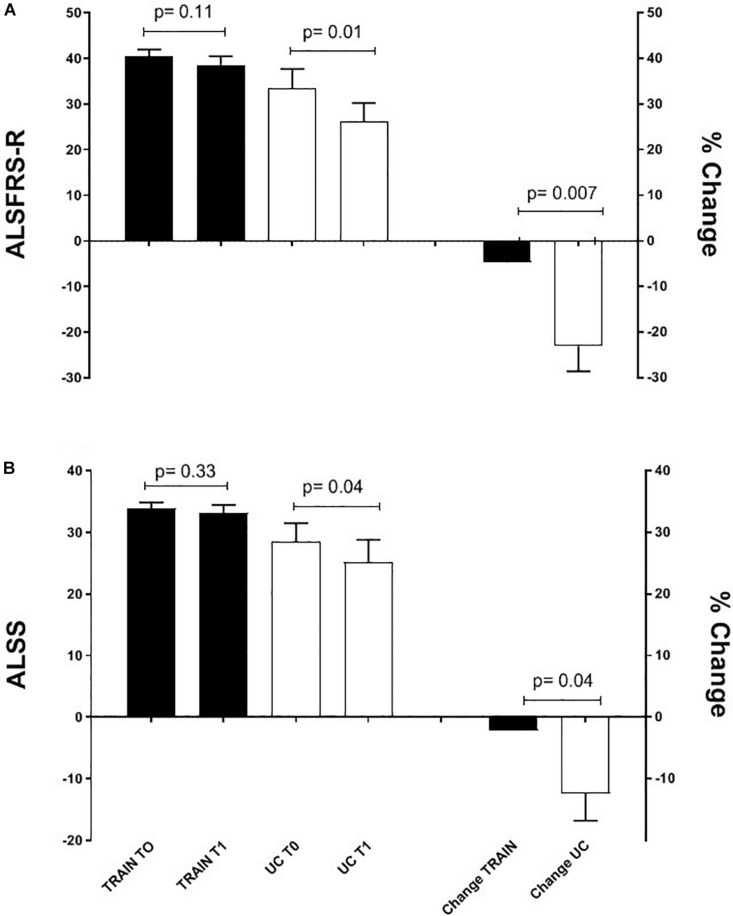
Mean ALSFRS-R **(A)** and ALS-SS **(B)** scores before (T0) and after 12 weeks (T1) of exercise training (TRAIN) and usual care (UC), and mean changes expressed as a percentage. Average data are expressed as mean + SE.

The ALSFRS-R and ALS-SS scores decreased significantly in UC by 23.0 ± 5.6% (*p* = 0.01, ES = 0.80) and by 12.4 ± 4.4% (*p* = 0.04, ES = 0.49) (Figure 5). In the TRAIN group there was not a significant reduction of these scores (−4.7 ± 2.6%, *p* = 0.11, ES = 0.41, and −2.2 ± 2.1%, *p* = 0.33, ES = 0.23, for the ALSFRS-R and ALS-SS, respectively). The changes in percentage of the ALSFRS-R and ALS-SS scores between the TRAIN and UC groups were significantly different (*p* = 0.007, ES = 2.04 and *p* = 0.04, ES = 1.4).

**FIGURE 6:**
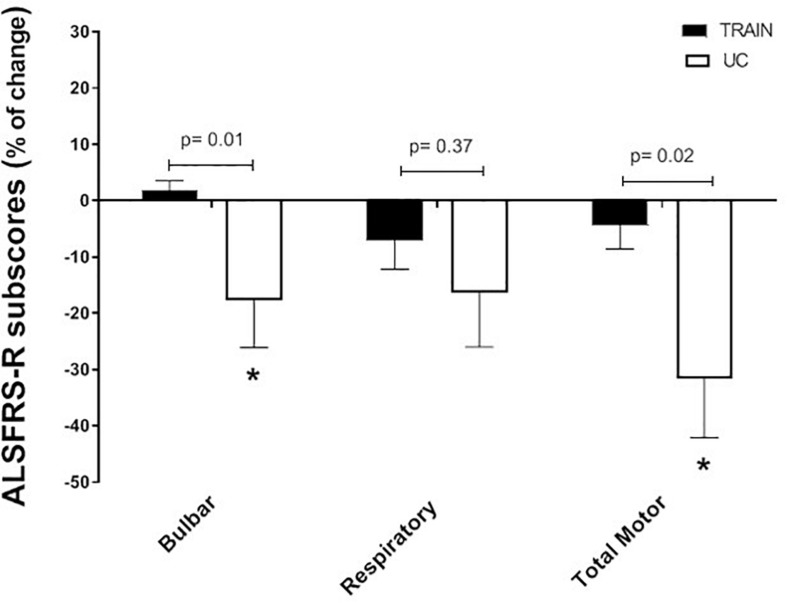
Changes in percentage for the ALSFRS-R subscores (bulbar, respiratory and total motor function) for the training (TRAIN) and usual care (UC) groups. Average data are expressed as mean + SE. ^∗^*p* < 0.05 between T0 and T1 in the UC group.

**FIGURE 7:**
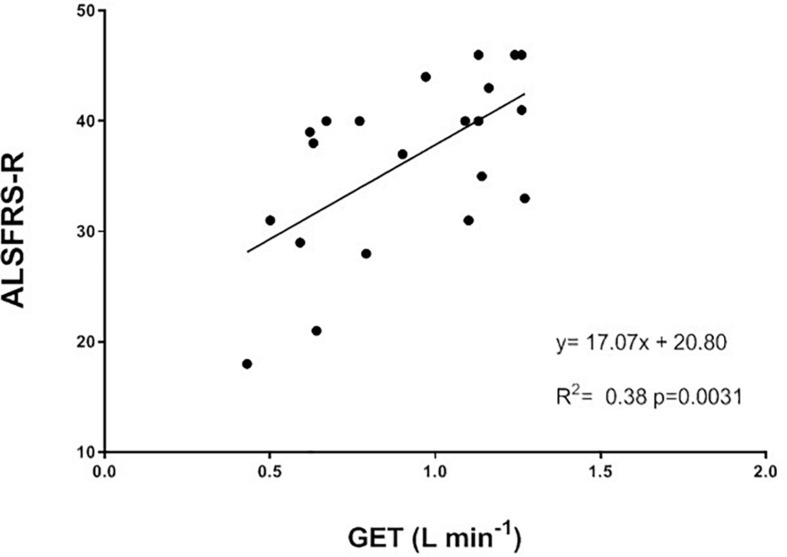
Correlation between gas exchange threshold (GET) and the ALS functional rating scale revised (ALSFRS-R) for all the participants in this study (TRAIN and UC groups).

When separating the ALSFRS-R results into the single sub-scores (bulbar, respiratory and total motor functions), the changes in percentage between the TRAIN and UC groups were significantly different for bulbar (*p* = 0.01) and total motor (*p* = 0.02), with UC showing greater decreases in these sub-scores (Figure 6). In UC, the total motor sub-scores decreased significantly by 31.6 ± 10.5% from 13.6 ± 2.5 to 10.2 ± 2.7 (*p* = 0.02), while in the TRAIN the decrease was only of 4.4 ± 4.1% (*p* = 0.25). When considering all the data from the TRAIN and UC groups, we found that the GET was significantly correlated to ALSFRS-R score, indicating that higher GETs in individuals with ALS are associated with higher functional score in the ALSFRS-R (Figure 7). QoL was not significantly different in the TRAIN and UC groups at T0 (7.0 ± 1.1 vs. 8.0 ± 1.2), but while the value was maintained in the TRAIN group at T1 (7.0 ± 0.8, *p* = 0.99), in UC the value showed a tendency to be lower (5.5 ± 1.5, *p* = 0.09).

## Discussion

This study shows that a combined moderate aerobic and strength training program is not only safe and enjoyable for participants, but also has beneficial effects on physical function (as measured by the TUG test) and aerobic fitness (as measured by GET). These positive effects were translated to the maintenance of the ALSFRS-R and ALSS scores in individuals with ALS who were training; on the contrary, the same scores decreased significantly in those who continued their standard of care.

The GET represents the point at which the energy requirement to sustain a determined effort cannot be fulfilled only by aerobic metabolism but there is also the need to use the anaerobic metabolism, with the consequent production of lactate and the development of muscle fatigue. The submaximal (at 80% of GET) aerobic exercise on the bicycle three times/week for 12 weeks had the effect to elevate the GET, which could improve the ability to sustain submaximal daily activities, such as walking or gardening, at a higher intensity or for longer time than before the training, and with less fatigue. This result suggests that part of the impaired aerobic capacity was not only due to the disease itself, but also to the muscle disuse, and that the right dose of exercise could help to restore (at least in part) the reduced aerobic fitness. Interestingly, Figure 4A shows a significant correlation between GET and 6MWT, but the improvement in aerobic capacity (as shown by GET) was not translated in a higher distance covered during the 6MWT after training. Because the 6MWT is also significantly correlated to the V̇O_2__peak_ (Figure 4B), the effect of an aerobic exercise with an intensity higher than that used in our study (around GET), may have a greater effect on this performance. This hypothesis should be investigated in future studies, without forgetting to ensure the right balance between exercise intensity and safety, and considering the high level of fatigue experienced by this population. The 13% non-significant increase in V̇O_2__peak_ in a population comprising very heterogeneous sub-entities, which carry the same expression (the ALS phenotype) but harbor widely different activated pathways, should be considered representative of a real positive change in the maximal aerobic capacity due to the training, particularly if compared to the 9% decrease in the UC group. Indeed, in patients with multiple sclerosis (another fragile population), changes in V̇O_2__peak_ were considered reliable if they were >10% (Langeskov-Christensen et al., 2014).

Even if not significant, due in part to the differential drop-outs and the intrinsic heterogeneity of the disease, it is interesting to note the direction of the arrows toward an increase in TRAIN and a decrease in UC for most of the physiological parameters evaluated during the CPET (Table 2). In particular, the tendency toward a significantly different change in the power output between TRAIN and UC seems due to the decrease (∼20%) of this parameter in the UC group. Although we cannot quantify how much of this decrease can be attributed to the disease and/or the disuse, our data support the hypothesis that tailored exercise training is able to counteract this reduced performance whatever the cause (as shown by a 3% increase of this parameter in the TRAIN group). The tendency toward a significantly different change in the peak ventilation between TRAIN and UC seems particularly due to an improved respiratory mechanics (∼27%) in the TRAIN group. A previous study by our group (Lanfranconi et al., 2017) has shown that the decrease in maximal ventilation in patients with ALS depends principally on the inability to increase the tidal volume, because of the weakness/fatigability of the respiratory muscles in individuals with ALS. The tendency toward a significant increase in peak ventilation after 12 weeks of moderate exercise training seems due to the concurrent increase in tidal volume (∼9%) and respiratory frequency (∼11%), and suggests that the submaximal aerobic training can maintain or even improve the efficiency of the respiratory muscles in supporting the O_2_ requirement during an intense exercise. Further studies, focusing on the effects of submaximal aerobic exercises on specific respiratory functions, should be performed in the future to reveal if this kind of training could provide an effective stimulus to the respiratory muscles of individuals with ALS.

The real challenge of an exercise training program for any clinical population is to produce an effect not only on the capacities that are specifically trained (i.e., strength, aerobic capacity, etc.), but to have an effect on more complex movements that incorporate different capacities and that define the ability to maintain physical independence. The maintenance of the ability to perform the TUG test (which incorporates strength, balance, and mobility) indicates the effectiveness of this type of exercise training in attenuating the decrease in physical function in individuals with ALS. Even if the changes in strength of the knee extensor muscles were not significantly different between TRAIN and UC, when translated to more complex movements related to the daily life activities, and associated probably to an improvement in balance and mobility (that was not tested in this project), there was a positive impact in maintaining physical function.

The beneficial impact of exercise training on physical function is also demonstrated by the attenuated reduction of the ALSFRS-R scores in TRAIN with respect to UC. The significant relationship between GET and the ALSFRS-R suggests that efforts should be directed toward trying to maintain the aerobic fitness in patients with ALS, which in turn will ensure the maintenance of physical independence for the longest possible time. Our results are in agreement with other reports that evaluated the impact of different type of exercise training on ALSFRS in individuals with ALS (Drory et al., 2001; Dal Bello-Haas et al., 2007; Lunetta et al., 2016). While Drory et al. (2001) reported only the total ALSFRS score (significant less decrease after 3 months of light training), Dal Bello-Haas et al. (2007) also presented the values of the motor ALSFRS sub-score. They found significant differences between a resistance exercise training group and a usual care group after 3 months (only motor ALSFRS sub-score) and 6 months (both total and motor ALSFRS scores) of intervention. Similar to our study, Lunetta et al. (2016) presented the results for the total ALSFRS score plus each sub-score (bulbar, motor, and respiratory). They showed a significantly higher score in the total ALSFRS after 180 days, but not after 90 days. While they did not find any difference in the respiratory and bulbar sub-scores, they found that the exercise training had its major effect on the motor domain. Interestingly, the exercise training proposed to our participants had an effect on not only the motor function (as expected) but also the bulbar function (not expected). The latter effect seems promising considering the difficulties faced by patients with a bulbar phenotype and the poorer outcomes reported. The ALS-SS scale confirmed, in a more articulated manner, the results found by the ALSFRS-R scale and supports the idea that exercise attenuates the disease-induced reduction in physical function in different areas of daily living in individuals with ALS.

The role of physical activity in improving the QoL has been demonstrated in various diseases. Recently, a meta-analysis conducted in six chronic brain disorders (Parkinson’s disease, Alzheimer’s disease, Huntington’s disease, multiple sclerosis, schizophrenia, and unipolar depression) confirmed that exercise is superior to pharmacological treatment in improving QoL (Dauwan et al., 2019). The maintenance of QoL found in this study in individuals with ALS who exercised, and the tendency toward lower values of QoL in individuals with ALS who did not exercise, is probably due to the fact that participants in the TRAIN group felt that their motor skills were maintained and that they could do something about it. In fact, the reduced QoL has been linked particularly to the physical status and anxiety of individuals with ALS (Young et al., 2019). In a disease where there is no cure and the only pharmacological treatment has limited efficacy, the exercise training represented a viable therapy.

### Participation

The differential drop-outs in TRAIN and UC could have biased our results (Bell et al., 2013), as some data could be lost due to non-random effects (i.e., patients withdraw for reasons related to their disease or treatment), particularly as patients with lower physical function or QoL values are more likely to drop out so as to leave the stronger patients in the UC group. Interestingly, the higher drop-out rate was in the UC and not in the TRAIN group, which was more challenged (e.g., travel time, the availability of a caregiver to take the participants to the gym) by having to travel three times/week for 12 weeks to the Clinica San Pietro where the gym was located. The high satisfaction in the exercise training program showed by the VAS (∼9), the high adherence by the individuals in the TRAIN group (∼85%), and the lower drop-outs indicate that the patients enjoyed the training program and the relationship with the human resources involved.

## Conclusion

Despite the small number of patients, this study supports the idea that tailored moderate-intensity exercise is not detrimental for patients with ALS and can counteract muscle disuse. The idea of non-exercising as a safe recommendation for individuals with ALS should be reconsidered in the light of these and other authors’ results. ALS care requires an integrated approach, and our results indicate that moderate-intensity exercise training strictly monitored by clinical exercise physiologists represents a real opportunity to maintain physical function and QoL in individuals with ALS, and should be introduced in the usual management and care of this disease.

## Members of the ME&SLA Study Group

Francesco Badi, Manuel Bianchi, Andrea Della Valentina, Marco Grandini, Carolina Lavazza, Alberto Maggiani, Laurent Mapelli, Valeria Milano, Giovanni Prochilo, Silvia Sosio (Italian Academy of Osteopathic Medicine, AIMO, Saronno, Italy); Annalisa Bosio, Diletta Cereda, Carlo Ferrarese, Laura Marzorati, Ornella Mauri, Paola Prometti (San Gerardo Hospital, Monza, Italy); Christian Lunetta (NEuroMuscular Omnicentre, NEMO, Fondazione Serena Onlus, Milan, Italy); Vittorio Mantero, Andrea Rigamonti Andrea Salmaggi (Alessandro Manzoni Hospital, Lecco, Italy); Nilo Riva (San Raffaele Hospital, Milan, Italy); Vincenzo Silani (University of Milan, Milan, Italy); Caterina Salito, Barbara Uva, Dario Bovio (Biocubica s.r.l., Milan, Italy).

## Data Availability Statement

The raw data supporting the conclusions of this article will be made available by the authors, without undue reservation, to any qualified researcher.

## Ethics Statement

The studies involving human participants were reviewed and approved by the University of Milano-Bicocca Ethics Committee. The patients/participants provided their written informed consent to participate in this study.

## Author Contributions

AF, FL, and LT contributed to the conception and design of the manuscript. AF, FL, GC, RB, SM, and LT contributed to the collection and analysis of the data. AF, FL, GC, RB, and SM contributed to the training of the participants. AM contributed to the provision of the study materials.

## Conflict of Interest

The authors declare that the research was conducted in the absence of any commercial or financial relationships that could be construed as a potential conflict of interest.
